# Early intervention in knee osteoarthritis with genicular artery embolization is associated with improved clinical outcomes

**DOI:** 10.1007/s00330-025-11702-1

**Published:** 2025-05-29

**Authors:** Tyler Callese, Lucas R. Cusumano, Hiro Sparks, Kara Masterson, Scott Genshaft, Jessica K. Stewart, Siddharth A. Padia

**Affiliations:** Division of Interventional Radiology, University of California Los Angeles, Los Angeles, CA USA

**Keywords:** Osteoarthritis, Embolization, Interventional radiology, Pain management

## Abstract

**Objectives:**

Genicular artery embolization (GAE) is an emerging, minimally invasive treatment option for knee OA (osteoarthritis). The purpose of this study was to analyze factors associated with clinical success in GAE.

**Materials and Methods:**

This IRB-approved, retrospective, single-center study analyzed 236 patients who underwent GAE for knee OA between May 2018 and September 2022. Clinical, radiographic, and technical variables were analyzed for association with clinical outcomes in GAE. The primary endpoint was clinical success assessed at one year.

**Results:**

Overall clinical success was reported in 128 patients (54.2%). Clinical success was observed in younger patients (67.5 ± 9.4 vs 71.5 ± 11.1 years, *p* < 0.01) with higher BMI (29.7 ± 5.3 vs 27.6 ± 6.4 kg/m^2^, *p* < 0.01). Lower KL grade (2.8 ± 0.8 vs 3.2 ± 0.8, *p* < 0.01), OARSI medial joint space narrowing grade < 3 (77.9% vs 63.6%, *p* < 0.01), and sparing of the descending genicular artery (53.9% vs 35.2%, *p* < 0.01) were associated with clinical success. Factors correlated with clinical success in multivariate logistic regression included: BMI (OR = 1.1, *p* = 0.03), KL grade (OR = 0.6, *p* < 0.01), and sparing of the descending genicular artery (OR = 2.4, *p* < 0.01).

**Conclusion:**

GAE is an effective procedure with durable clinical improvement at one year, particularly in younger patients and those with mild-to-moderate knee osteoarthritis. These findings suggest that early intervention with GAE may be appropriate in patients who are not candidates for, or do not desire, total knee arthroplasty.

**Key Points:**

***Question*** *GAE is a minimally invasive intervention for knee osteoarthritis, however, there is a paucity of evidence describing factors associated with improved clinical outcomes*.

***Findings*** *Factors associated with clinical success in GAE included younger patients and those with early-stage osteoarthritis*.

***Clinical relevance*** *These findings support GAE as an early intervention for patients with symptomatic knee osteoarthritis*.

**Graphical Abstract:**

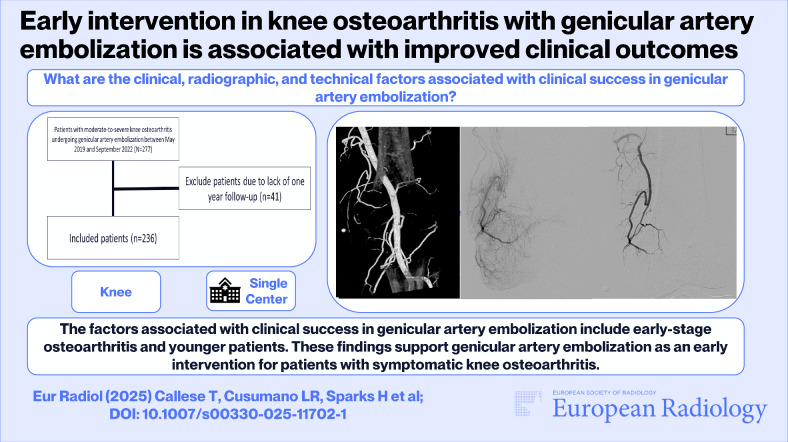

## Introduction

Knee osteoarthritis (OA) is a leading cause of disability and chronic pain in the United States, with a lifetime prevalence of approximately 44.7% [1, 2]. The burden of disease and associated healthcare costs are expected to increase as the population continues to age [2].

The current spectrum of OA treatment ranges from non-surgical therapies to total knee arthroplasty (TKA). Conservative therapies, including lifestyle modifications, physical therapy, anti-inflammatory medications, and intra-articular injections, are limited in efficacy and durability [3]. TKA is the established gold standard treatment for knee OA, with approximately 790,000 procedures performed in the United States annually and an increasing number being performed in younger patients [4, 5]. TKA has notable drawbacks. These include the need for joint revisions within 10–20 years, which limits its use in younger patients, and rates of dissatisfaction up to 21% following the procedure [6]. Additionally, the intensive rehabilitation and recovery time required for a successful outcome may also serve as a deterrent for many people. Consequently, there is a significant treatment gap for patients who have exhausted non-surgical therapies yet are not candidates for, or do not wish to undergo, knee replacement.

Genicular artery embolization (GAE) is a minimally invasive therapy poised to serve as a possible bridging or complementary therapy between conservative treatments and TKA [7–12]. GAE is an outpatient procedure performed under minimal or moderate sedation with minimal risks [7]. Several trials have demonstrated GAE to be safe and potentially effective in the treatment of symptomatic knee OA. However, there is a paucity of evidence guiding patient selection [13, 14]. Success rates range from 60% to 86%, suggesting that a significant percentage of patients are non-responders to GAE [15, 16].

The purpose of this study was to determine the clinical, radiographic, and technical factors associated with clinical success in GAE for symptomatic knee OA.

## Materials and methods

### Study design

This single-center retrospective study was approved by the institutional review board and informed consent was waived.

### Patients

**Fig. 1:**
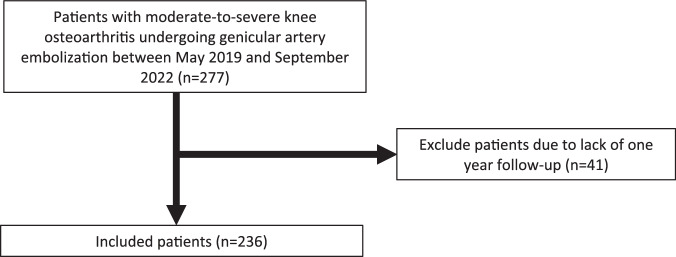
Study design flow diagram

All patients who underwent GAE for symptomatic knee OA between May 2018 and September 2022 were reviewed (Fig. 1). Inclusion criteria included ineligibility for or refusal of TKA, moderate to severe knee pain defined as pain greater than or equal to 5 out of 10 on the visual analog scale, Kellgren–Lawrence (KL) grade 2–4 radiographic knee OA, and failure of conservative management. Failure of conservative management included no clinical improvement with physical therapy, medical management, and/or intra-articular injections. Exclusion criteria included iodinated contrast allergy, impaired renal function, and a follow-up period of less than one year. One-year follow-up effectively captures long-term outcomes to assess for durability and avoids capturing temporary benefits (i.e., placebo effect) that can affect short-term follow-up assessments.

### Procedures

GAE procedures were performed at a single academic tertiary care center in the ambulatory setting under moderate sedation by one of three board-certified interventional radiologists using a previously described technique [7].

**Fig. 2:**
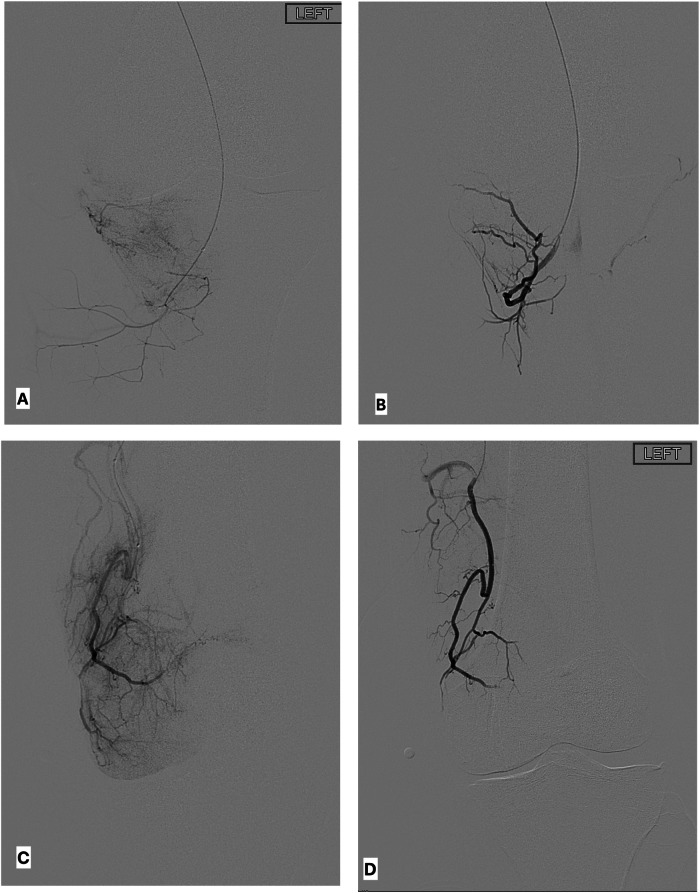
Intra-procedural imaging during GAE, continued. **A** Inferior medial genicular artery DSA demonstrates abnormal vascular blush along the inferior medial joint space. **B** Post-embolization inferior medial genicular artery DSA demonstrates successful pruning of the abnormal hyperemia with expected patency of the normal vessels. **C** Descending genicular artery (DGA) DSA demonstrates abnormal hyperemia along the superior medial joint space. **D** Post-embolization DGA DSA demonstrates successful pruning of the abnormal hyperemia with expected patency of the normal vessels

Briefly, radiopaque skin markers were placed at the site(s) of knee pain prior to the procedure. Ipsilateral or contralateral common femoral artery access was performed with a preference for ipsilateral antegrade access due to shorter working lengths and increased dexterity of microcatheters and microwires. A digital subtraction angiography (DSA) from the superficial femoral artery with the treated knee at the isocenter was performed. Prolonged DSA was performed to identify areas of abnormal synovial hyperemia or vascular staining. This was followed by contrast-enhanced cone-beam computed tomography to aid in the identification of genicular anatomy and target vessels [17]. Target genicular arteries were subsequently catheterized using a 1.7–2.4 Fr microcatheter. Embolization was performed with 100-mm particles (Embozene, Varian Medical Systems). The embolization endpoint was pruning of abnormal hyperemic vascular blush while preserving the normal arterial flow (Fig. 2). Following embolization of all target arteries, hemostasis was achieved with manual compression for antegrade access or a vascular closure device for contralateral access. Patients were discharged 4 h following the procedure.

### Patient factors

**Fig. 3:**
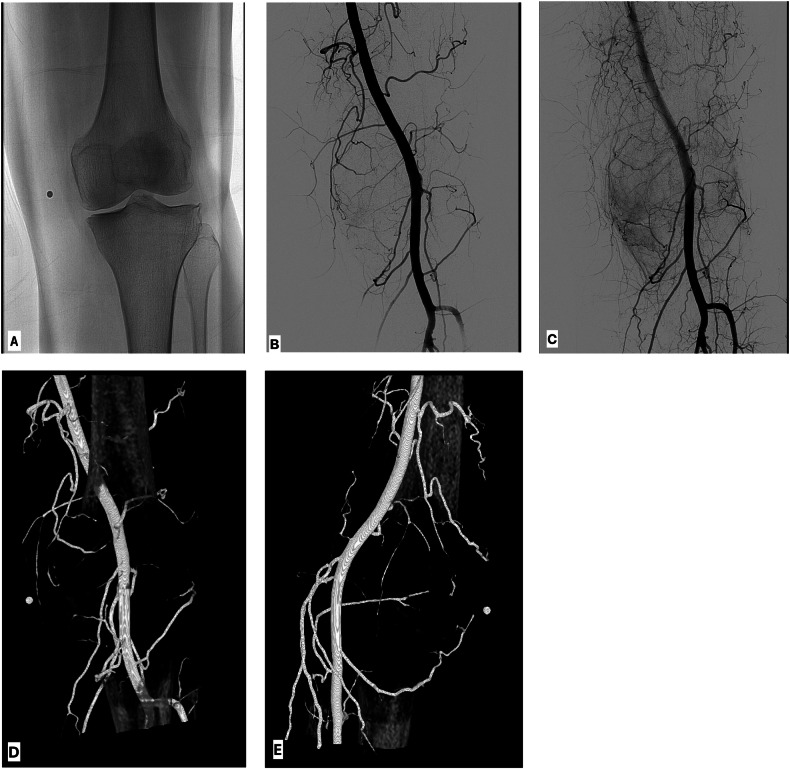
Intra-procedural imaging during GAE. **A** Scout radiograph demonstrated moderate medial joint space narrowing and mild endplate changes (KL grade 3). A radiopaque marker identifies the area of pain along the medial joint space. **B** DSA from the superficial femoral artery. **C** Delayed images from DSA demonstrate hypervascularity along the medial joint space corresponding to the area of pain. **D** Three-dimensional AP and (**E**) lateral reconstructions from intra-procedural cone beam CT demonstrate branches of the DGA and inferior medial genicular artery coursing towards the area of pain along the medial joint space, as indicated by radiopaque marker

Variables included age, weight, body mass index (BMI), location of pain, number of symptomatic sites, and symptomatic quadrant location. Symptom location was identified during the procedure by radiopaque markers placed on the skin corresponding to the patient’s reported site of pain (Fig. 3).

### Imaging factors

The radiographic variables assessed included the presence of a knee joint effusion, significant arterial atherosclerosis, KL grade, and Osteoarthritis Research Society International (OARSI) joint space narrowing grade. Knee effusions were identified on pre-operative imaging, while atherosclerosis was identified on intra-procedural DSA.

Pre-procedural radiographs were independently evaluated by two radiologists for KL and OARSI grading, with any discrepancies resolved through consensus. KL grading is the most widely used radiologic classification system for OA screening and grading of overall clinical severity [18]. OARSI provides a more granular evaluation of joint changes and includes a radiographic atlas for reference [19]. Comprehensive OARSI classification includes evaluation of marginal osteophytes in four locations, osseous attrition/sclerosis in three locations, and joint space narrowing in three compartments. Patellofemoral compartment joint space narrowing is described in a prior version of the OARSI grading system [20]. Compartment-specific joint space narrowing grades were included in this study as it was felt to accurately reflect the severity of disease within individual compartments and associated abnormal vascularity [19].

### Technical factors

Variables included the total number of vessels embolized, specific arteries embolized, and patient response to intra-arterial nitroglycerin within the genicular artery. The total number of arteries embolized was included as it has been previously suggested that embolization of more than one vessel is required to completely address robust arterial anastomoses around the knee [15]. This rich anastomotic network becomes more prominent after the dominant genicular artery supplying the symptomatic location is embolized [15]. After catheterization of a target genicular artery, 100 mg of nitroglycerin was administered through the microcatheter to prevent or decrease vasospasm caused by manipulation and embolization of the small genicular arteries and improve visualization of the vessels [21]. Additionally, reproduction of pain following nitroglycerin administration in the symptomatic quadrant was noted.

### Outcome measures

The primary outcome was clinical success at one year using the Western Ontario and McMaster Universities Osteoarthritis Index (WOMAC) [22]. WOMAC is a self-administered, OA-specific questionnaire assessing pain, stiffness, and physical function in the affected knee. Clinical success was defined as ≥ 50% improvement in WOMAC score at one year (WOMAC 50). While the minimum clinically important difference has been reported at less than 20% improvement in previous studies, a 50% reduction in WOMAC was chosen in this study to identify those with significant improvement in pain, which would likely be unrelated to any potential placebo effect. Secondary outcomes included identification of clinical, radiographic, and technical factors associated with clinical success, as described above.

### Statistical analysis

For the primary analysis, clinical success was summarized with counts and proportions. Comparisons between clinical success and failure groups were performed using Wilcoxon rank sum tests for continuous variables and Chi-square tests for categorical variables. Multivariate logistic regression was performed to assess for confounding interactions and simultaneous effects of variables that were significantly different between groups on univariate analysis. *p*-values less than 0.05 were considered significant. All statistical analysis was performed with software R (R version 2023.06.1 + 524).

## Results

A total of 277 patients underwent GAE between May 2018 and September 2022. 236 of 277 (85%) patients met inclusion criteria (Fig. 1). Overall clinical success, defined as ≥ 50% decrease in WOMAC at one year, was 54.2% (128 out of 236 patients).

Baseline demographic and patient factors are summarized in Table 1. There was a significant between-group age difference with younger patients associated with clinical success (67.5 years vs 71.5 years, *p* = 0.003) and those with a higher BMI (29.7 vs 27.7 kg/m^2^, *p* = 0.008). There was no difference in clinical success based on the location of pain or a number of symptomatic locations.

**Table 1:** Baseline demographics and clinical factors associated with clinical success in patients who underwent GAE for knee osteoarthritis

	All (*N* = 236)	Success (*N* = 128)	Failure (*N* = 108)	*p*-value
Age (years, mean, range)	69.3 (40–95)	67.5 (40–90)	71.5 (46–95)	0.003^*,1^
Weight (kg, mean, range)	81.5 (44–164)	83.1 (44–164)	79.5 (47–137)	0.11^1^
Height (cm, mean, range)	168.1 (142–196)	167.3 (142–196)	169.1 (147–193)	0.21^1^
Body mass index (kg/m^2^, mean, range)	28.8 (18.3–54.9)	29.7 (18.3–54.9)	27.7 (18.3–45.8)	0.008^*,^^1^
Treated side				0.86^2^
Left	126 (53.4%)	69 (53.9%)	57 (52.8%)	
Right	110 (46.6%)	59 (46.1%)	51 (47.2%)	
Symptomatic sites marked (number)				0.80^2^
1	142 (60.2%)	76 (59.4%)	66 (61.1%)	
2	63 (26.7%)	34 (26.6%)	29 (26.9%)	
3	27 (11.4%)	16 (12.5%)	11 (10.2%)	
4	1 (0.4%)	1 (0.8%)	0 (0%)	
Symptomatic site				
Medial	104 (44.1%)	59 (46.1%)	45 (41.7%)	0.50^2^
Lateral	185 (78.4%)	99 (77.3%)	86 (79.6%)	0.67^2^
Superior midline	154 (65.3%)	86 (67.2%)	68 (63.0%)	0.50^2^
Inferior midline	118 (50.0%)	65 (50.8%)	43 (49.1%)	0.794^2^

^*^ *p* < 0.05

^1^ Wilcoxan Rank-Sum

^2^ Chi-square

Radiographic factors are summarized in Table 2. Lower KL grade (2.8 ± 0.8 vs 3.2 ± 0.8, *p* < 0.01) and OARSI medial joint space narrowing grade < 3 (77.9% vs 63.6%, *p* < 0.01) were associated with clinical success. There was no difference in clinical outcomes based on knee effusion, atherosclerosis, or lateral or patellofemoral compartment joint space narrowing.

**Table 2:** Radiographic factors associated with clinical success in patients who underwent GAE for knee osteoarthritis

	All	Success	Failure	*p*
Knee effusion (*N* = 236)	188 (79.7%)	99 of 128 (77.3%)	89 of 108 (82.4%)	0.34
Atherosclerosis (*N* = 207)	24 of 207 (11.6%)	11 of 112 (9.8%)	13 of 95 (13.7%)	0.39
KL grade	*N* = 234	*N* = 127	*N* = 107	0.01^*^
1	10 (4.3%)	7 (5.5%)	3 (2.8%)	
2	58 (24.8%)	38 (29.9%)	20 (18.7%)	
3	96 (41.0%)	55 (43.3%)	41 (38.3%)	
4	70 (29.9%)	27 (21.3%)	43 (40.2%)	
OARSI^1^ joint space narrowing grade				
Medial compartment				0.014^*^
1	69 (29.5%)	46 (36.2%)	23 (21.5%)	
2	98 (41.9%)	53 (41.7%)	45 (42.1%)	
3	67 (28.6%)	28 (22.0%)	39 (36.4%)	
Lateral compartment				0.31
0	37 (15.8%)	24 (18.9%)	13 (12.1%)	
1	116 (49.6%)	65 (51.2%)	51 (47.7%)	
2	62 (26.5%)	29 (22.8%)	33 (30.8%)	
3	19 (8.1%)	9 (7.1%)	10 (9.3%)	
Patellofemoral compartment				0.41
0	19 (8.1%)	12 (9.4%)	7 (6.5%)	
1	128 (54.7%)	70 (55.1%)	58 (54.2%)	
2	60 (25.6%)	28 (22.0%)	32 (29.9%)	
3	27 (11.5%)	17 (13.4%)	10 (9.3%)	

^*^ *p*-value < 0.05

^1^ OARSI

Technical factors are summarized in Table 3. Sparing of the descending genicular artery (DGA) during embolization was associated with clinical success (53.9% vs 35.2%, *p* < 0.01). There was no significant difference with regard to the total number of arteries embolized or embolization of any other specific genicular arteries.

**Table 3:** Technical factors associated with clinical success in patients who underwent GAE for knee osteoarthritis

	All (*N* = 236)	Success (*N* = 128)	Failure (*N* = 108)	*p*
Number of arteries embolized				0.32
1	83 (35.2%)	50 (39.1%)	33 (30.6%)	
2	117 (49.6%)	63 (49.2%)	54 (50.0%)	
3	29 (12.3%)	12 (9.4%)	17 (15.7%)	
4	7 (3.0%)	3 (2.3%)	4 (3.7%)	
Arteries embolized				
Descending genicular	129 (54.7%)	59 (46.1%)	70 (64.8%)	0.004^*^
Superior medial	54 (22.9%)	31 (24.2%)	23 (21.3%)	0.59
Superior lateral	64 (27.1%)	32 (25.0%)	32 (29.6%)	0.43
Inferior medial	79 (33.5%)	45 (35.2%)	34 (31.5%)	0.55
Inferior lateral	91 (38.6%)	50 (39.1%)	41 (38.0%)	0.86
Recurrent tibial	8 (3.4%)	4 (3.1%)	4 (3.7%)	0.81
Superior patellar	8 (3.4%)	4 (3.1%)	4 (3.7%)	0.81
Response to NTG^1^ (*N* = 199)	108 (54.3%)	57 of 102 (55.9%)	51 of 97 (52.6%)	0.64

^*^ *p*-value < 0.05

^1^ NTG, intra-arterial nitroglycerine

Factors predicting clinical success are summarized in Table 4. Age trended toward clinical significance with an odds ratio (OR) of success of 0.97 for each incremental year increase in age. The OR of clinical success for each whole integer increase in BMI was 1.06 (*p* = 0.025), 0.6 (*p* = 0.004) for each incremental increase in KL score, and 2.4 (*p* = 0.002) when the DGA was not embolized.

**Table 4:** Multivariate logistic regression for 1-year clinical success, (*N* = 234)

Predictors	OR	95% Confidence interval	*p*-value
Age	0.97	0.94–1.0	0.066
Body mass index	1.06	1.01–1.12	0.025^*^
KL grade	0.6	0.42–0.85	0.004^*^
Sparing DGA	2.4	1.39–4.34	0.002^*^

Input variables were selected based on univariate performance

^*^ *p*-value < 0.05

## Discussion

GAE is a safe and effective minimally invasive therapy for symptomatic knee OA poised to address the current OA treatment gap between conservative nonsurgical care and TKA. The lack of data informing optimal patient selection has led to a wide range in reported response rates [7–11]. The purpose of this study was to determine the clinical, radiographic, and technical factors associated with clinical success in GAE. The factors predictive of clinical success included younger patient age, higher BMI, lower KL grade, and sparing of the DGA during embolization.

Younger patients with symptomatic knee OA present a clinical challenge because they are often more physically active and quickly exhaust non-invasive therapies [23]. While TKA may be an option for these patients, most patients prefer delaying TKA for as long as possible due to the significant rehabilitation process and the need for subsequent revision surgery in 10–20 years [24]. The results of the present study suggest that younger patients would be ideal candidates for GAE. While older age was associated with a greater risk of treatment failure on univariate analyses, this relationship did not reach statistical significance when controlling for confounding variables on multivariate analysis. This is likely related to the collinearity between age and KL score, where older patients tend to have worse radiographic OA, and points toward OA severity as the underlying driver of GAE outcomes. Still, the trend toward statistical significance on multivariate analysis (*p* = 0.066), suggests that younger age independently correlates with GAE success.

This study found higher BMI to be predictive of clinical success. Larger studies have demonstrated that a BMI > 30 more than doubles the risk of symptomatic knee OA and synovitis [25, 26]. In addition to effects related to greater mechanical stress on the knee, adipose tissue secretes adipokines that are key regulators of inflammation and angiogenesis [27–29]. Multiple prior studies have demonstrated direct associations between obesity-induced adipokine production and the onset of OA [30, 31]. In particular, OA in obese patients is associated with an enriched population of synovial lymphocytes compared with healthy controls [29]. Given that GAE specifically targets the inflammatory rather than the mechanical degenerative axis of OA, the results of the present study indicate that higher BMI patients are particularly well served by GAE. The interplay between immune cells, inflammatory mediators, and direct mechanical wear points toward obesity as a key factor in ongoing GAE research.

The results of the current study found that GAE is less effective as joint space degeneration progresses. For each increase in KL grade, the OR of clinical success was 0.6 (*p* = 0.004). These results are similar to those previously reported [13, 16, 32]. Decreased clinical success with severe OA is possibly due to the increased mechanical pain component (direct bone-on-bone contact) as opposed to the predominant inflammatory synovial pain in patients with mild-to-moderate radiographic OA [13]. This supports the use of GAE in patients with mild-to-moderate radiographic OA changes and aids in addressing the current treatment gap for patients with symptomatic knee OA who are not candidates for, or wish to defer, TKA.

Sparing of the DGA was associated with improved clinical success with an OR of 2.4 (*p* = 0.004). Genicular artery anatomy is complex and highly variable [17, 33]. The most variable anatomy is seen in the DGA, where its “normal” anatomy, consisting of a superficial myocutaneous branch and a separate deep synovial branch, is only seen in 77% of patients [33]. The results of this study suggest that the DGA should only be embolized if it is the dominant artery supplying the area of abnormal vascularity and pain. Additionally, this finding suggests that the other genicular arteries supplying the medial knee (i.e., superior medial and inferior medial genicular arteries) should be embolized first, and the DGA should then be embolized if there is residual abnormal vascular blush.

Magnetic resonance imaging (MRI) of the knee plays an important role in the diagnosis of knee pathology. The importance and prognostic capacity of knee MRI in GAE is a current focus of investigation in several ongoing clinical trials.

This study has certain limitations. First, this is a retrospective review, and no control group was included. Second, adjunctive measures such as continued use of conservative therapies post-GAE were not controlled for, which may have influenced outcomes. Third, GAE was performed at a single center by three experienced interventional radiologists with a large GAE experience, which may limit the translation of results to other centers.

In conclusion, GAE for symptomatic knee OA is an effective procedure with high rates of clinical success at one year. Younger age, higher BMI, and mild-to-moderate radiographic OA are factors associated with clinical success. These findings aid in patient selection and support the inclusion of GAE as a minimally invasive therapy poised to address the current treatment gap in options for patients with symptomatic knee OA and mild-to-moderate radiographic changes refractory to conservative management and contraindication for TKA.

## Abbreviations

DGA: Descending genicular artery
DSA: Digital subtraction angiography
GAE: Genicular artery embolization
KL: Kellgren–Lawrence
OA: Osteoarthritis
OARSI: Osteoarthritis Research Society International
OR: Odds ratio
TKA: Total knee arthroplasty
WOMAC: Western Ontario and McMaster Universities Osteoarthritis Index
